# A randomized controlled trial of an intervention delivered by mobile phone app instant messaging to increase the acceptability of effective contraception among young people in Tajikistan

**DOI:** 10.1186/s12978-018-0473-z

**Published:** 2018-02-13

**Authors:** Ona McCarthy, Irrfan Ahamed, Firuza Kulaeva, Ravshan Tokhirov, Salokhiddin Saibov, Marieka Vandewiele, Sarah Standaert, Baptiste Leurent, Phil Edwards, Melissa Palmer, Caroline Free

**Affiliations:** 1 Department of Population Health, Faculty of Epidemiology and Population Health, London School of Hygiene & Tropical Medicine, Keppel Street, London, WC1E 7HT UK; 2 Tajik Family Planning Association, 10 Rudaki Avenue, TC ‘Sadbarg’, 7th floor, Dushanbe, Tajikistan; 3 International Planned Parenthood Federation European Network, Rue Royale 146, 1000 Brussels, Belgium; 4 Department of Medical Statistics, Faculty of Epidemiology and Population Health, London School of Hygiene & Tropical Medicine, Keppel Street, London, WC1E 7HT UK

**Keywords:** Randomized controlled trial, Tajikistan, Contraception, Smart phone, Reproductive health, Young adults

## Abstract

**Background:**

Unintended pregnancy is associated with poorer health outcomes for women and their families. In Tajikistan, around 26% of married 15–24 year old women have an unmet need for contraception. There is some evidence that interventions delivered by mobile phone can affect contraceptive-related behaviour and knowledge. We developed an intervention delivered by mobile phone app instant messaging to improve acceptability of effective contraceptive methods among young people in Tajikistan.

**Methods:**

This was a randomized controlled trial among Tajik people aged 16–24. Participants allocated to the intervention arm had access to an app plus intervention messages. Participants allocated to the control arm had access to the app plus control messages. The primary outcome was acceptability of at least one method of effective contraception at 4 months. Secondary outcomes were use of effective contraception at 4 months and during the study, acceptability of individual methods, service uptake, unintended pregnancy and induced abortion. Process outcomes were knowledge, perceived norms, personal agency and intention. Outcomes were analysed using logistic and linear regression. We conducted a pre-specified subgroup analysis and a post-hoc analysis of change in acceptability from baseline to follow-up.

**Results:**

Five hundred and seventy-three participants were enrolled. Intervention content was included on the app, causing contamination. Four hundred and seventy-two (82%) completed follow-up for the primary outcome. There was no evidence of a difference in acceptability of effective contraception between the groups (66% in the intervention arm vs 64% in the control arm, adjusted OR 1.21, 95% CI .80–1.83, *p* = 0.36). There were no differences in the secondary or process outcomes between groups. There was some evidence that the effect of the intervention was greater among women compared to men (interaction test *p* = 0.03). There was an increase in acceptability of effective contraception from baseline to follow-up (2% to 65%, *p* < 0.001).

**Conclusions:**

The whole intervention delivered by instant messaging provided no additional benefit over a portion of the intervention delivered by app pages. The important increase in contraceptive acceptability from baseline to follow-up suggests that the intervention content included on the app may influence attitudes. Further research is needed to establish the effect of the intervention on attitudes towards and use of effective contraception among married/sexually active young people.

**Trial registration:**

Clinicaltrial.gov NCT02905513. Date of registration: 14 September 2016.

**Electronic supplementary material:**

The online version of this article (10.1186/s12978-018-0473-z) contains supplementary material, which is available to authorized users.

## Plain English summary

Unintended pregnancy is associated with poor health and social outcomes for women and their families. Despite wide availability of contraception, many women globally face barriers in realizing their fertility desires. A woman has an unmet need for modern contraception if she wants to avoid a pregnancy but currently uses no method or a traditional method. In Tajikistan, unmet need for contraception is approximately 26% among married 15–24 year olds. Oppositional attitudes towards contraception (both their own and others’) is a common reason women provide for not using contraception.

We developed an intervention delivered by mobile phone to increase the acceptability of effective contraception among young people in Tajikistan. The intervention was developed with young people using an established approach grounded in behavioural science. We conducted a randomized controlled trial to evaluate the effect of the intervention on acceptability of effective contraception. Participants allocated to the intervention group had access to an app plus the intervention messages. Participants allocated to the control group had access to the app plus control messages. The app contained a proportion of the intervention messages that targeted knowledge of and attitudes towards effective contraception. This was different from what was planned in the trial protocol.

The intervention instant messages did not have an added benefit over the app with regards to any of the outcomes. When data from both groups were analysed together, there was a large increase in acceptability of effective contraception from baseline to follow-up (2% at baseline to 65% at follow-up). While we cannot attribute this increase unequivocally to the intervention content, it suggests that providing accurate information and targeting beliefs that influence contraceptive use may be sufficient in changing attitudes towards these methods among young people in Tajikistan. Further research is needed to reliably establish the effect of the intervention on attitudes towards and use of effective contraceptive methods among married/sexually active young people.

## Background

Unintended pregnancy persists as a global health problem, with people in lower income countries experiencing them at a higher rate [1]. Unintended pregnancy is associated with a multitude of negative health and economic outcomes for women and their families [2–11]. It is estimated that modern contraceptive use currently prevents 307 million unintended pregnancies each year in developing regions [12]. Satisfying unmet need for modern contraception in these regions would reduce unintended pregnancies by 74% [12]. A woman has an unmet need for modern contraception if she wants to avoid a pregnancy but currently uses no method or a traditional method [13].

Despite a number of governmental policy initiatives and strategies aimed at improving reproductive health in Tajikistan, young people in the country face challenges in gaining accurate information about contraception and in accessing services [14, 15]. The 2012 Tajikistan Demographic and Health Survey is the most reliable resource for family planning data in the country at present [16]. The survey estimates that Tajik women have an average of half a child more than their desired number, implying that if unintended pregnancies were avoided, the total fertility rate would be 3.3 births per woman rather than the actual 3.8 [16]. The effective contraceptive methods available in Tajikistan are oral contraceptive pills (OCs), intrauterine devices (IUDs), injectables and implants (‘effective methods are methods with a less than 10% typical use failure rate at 12 months [17–19]). Though these methods are available, around 26% of married 15–24 year old women have an unmet need for contraception [16]. Unmet need is the highest between the ages of 20 to 29 [20]. The main reason women with an unmet need provide for not using contraception are oppositional attitudes towards contraception, both their own and others’ [20]. The next common reasons relate to low perceived pregnancy risk and negative attitudes about the methods, such as fear of side-effects [20].

Over the past few decades, the dramatic global increase in mobile phone ownership has engendered enthusiasm amongst researchers and health care providers regarding the use of mobile phones for health care delivery [21–32]. Trials have provided some evidence that interventions delivered by mobile phone can improve contraceptive-related behaviours [33–36] and knowledge [37–39], however others have failed to find an effect [40–43]. The London School of Hygiene and Tropical Medicine (LSHTM) and the Tajik Family Planning Association (TFPA), a Member Association of the International Planned Parenthood Federation (IPPF) collaborated to develop and evaluate an intervention delivered by mobile phone to improve attitudes towards the effective contraceptive methods among young people in Tajikistan.

To evaluate the intervention, we conducted a randomized controlled trial from November 2016 to July 2017. This paper reports the results of the trial. To the best of our knowledge, this is the first trial to evaluate a contraceptive behavioural intervention delivered by mobile phone in Tajikistan. The results contribute to an understanding about how to help young people in Tajikistan avoid unintended pregnancies.

## Methods

The methods reported in this section were first published in the trial protocol [44] and the statistical analysis plan [45].

### Study design and participants

This was a parallel group, individually randomized superiority trial with a 1:1 allocation ratio. The aim of this trial was to assess the effect of the intervention on the acceptability of effective contraceptive methods among young people in Tajikistan. Participants were eligible to take part in the trial if they were between the ages of 16 and 24, owned a personal Android mobile phone, lived in Tajikistan, could provide informed consent and could read Tajik or Russian. Participants must also have been willing to download a mobile phone app and receive instant messages about contraception through the app. Participants provided informed consent though the secure online trial database and randomization system. All participants received usual care (the normal care that a young person would receive if they attended a sexual and reproductive health service in Tajikistan) and were free to seek any other support.

### Intervention and control

The intervention was developed with young Tajik people in 2015–2016 guided by an established approach grounded in behavioural science [46]. It consisted of short mobile phone instant messages delivered through TFPA’s ‘healthy lifestyles’ app over 4 months. It was informed by the Integrated Behavioural Model (IBM) [47] and contained 10 behaviour change methods (BCM) (belief selection, facilitation, anticipated regret, guided practice, verbal persuasion, tailoring, cultural similarity, arguments, shifting perspective and goal setting) [48], adapted for delivery by mobile phone. The messages provided information about contraception, targeted beliefs identified in the development phase that influence contraceptive use and aimed to support young people in believing that they can influence their reproductive health.

The messages are tailored according to marital status and gender, resulting in four sets of messages (female-married, female-not married, male-married and male-not married). The majority of the messages in the four sets are the same, with minor tailoring so that the messages are relevant to these groups. (Marital status was used as a proxy for sexual activity because the target group and TFPA considered it inappropriate to ask directly about sexual activity.) Further details about the intervention are presented in the trial protocol [44] and in a forthcoming intervention development publication.

#### Contamination

Participants allocated to the intervention arm had access to the app plus the intervention instant messages. Participants allocated to the control arm had access to the app plus control instant messages about trial participation. Contrary to what was planned in the trial protocol [44], the app contained intervention content. The app was intended to contain only basic information about contraception and no behaviour change methods. This contamination occurred due to a misunderstanding between the partners collaborating in the research.

The app contraception pages included just under a third of the intervention content. Specifically, 57% of the female-married intervention messages that provide accurate information about the effective contraceptive methods and 36% of the messages that use the BCM ‘belief selection’ were included on the app. Forty-four percent of the female-married intervention content included on the app used the same words as the intervention messages (56% did not use the same words but was very similar and conveyed the same meaning). The intervention content included on the app aimed to help individuals: name the effective methods, describe how the effective methods work, list services that provide effective contraception, list the risks and benefits of the effective methods, describe how methods are used, express positive attitudes towards the effective methods and differentiate between real potential side-effects and misconceptions about the methods.

### Allocation and intervention delivery

After providing informed consent, participants completed the baseline questionnaire through the database and randomization system. The allocation sequence was generated by the remote computer-based randomization software. Randomization occurred immediately after baseline data was submitted. All participants downloaded the app immediately after they submitted their baseline data. The delivery of the intervention (and control) instant messages began on the same day if participants downloaded the app before 13:00 and the following day if they downloaded it after 13:00.

### Protecting against bias

Due to the nature of the intervention, participants would have been aware of the allocation soon after they started receiving the messages. Local research staff collecting outcome data were masked to allocation unless the participant revealed it to them. Researchers that analysed the data were masked to treatment allocation.

### Outcomes

#### Primary outcome

The primary outcome was the proportion of participants reporting that at least one method of effective contraception was acceptable at 4 months post randomization. The primary outcome measure was constructed based on guidelines for measuring IBM constructs [47, 49, 50] and tested for face validity with the target group. The acceptability of each method was measured by the following stems: Using the [method] *…causes infertility, …causes unwanted side effects, …is easy, …is a good way to prevent pregnancy* and *I would recommend the [method] to a friend*. The IUD and implant include an additional stem: *The* [method] *insertion would not be a problem*. The response options for each scale were strongly disagree, disagree, not sure, agree, strongly agree and I do not know what the [method] is. A method was acceptable if participants reported ‘agree’ or ‘strongly agree’ for all scales except for ‘…causes infertility’ and ‘…causes unwanted side effects’ stems, for which ‘disagree’ or ‘strongly disagree’ indicated acceptability.

#### Secondary outcomes

Secondary outcomes were: use (or partner’s use) of effective contraception; acceptability of individual methods; use (or partner’s use) of effective contraception at any time during the 4 months; service uptake; unintended pregnancy and induced abortion.

##### Process outcomes

The process outcomes were: knowledge of effective contraception; perceived norms in relation to using and communicating with partners about contraception; personal agency in using (women only) and communicating with partners about contraception; intention to use effective contraception (women only) and intervention dose received. Details about the scales used to measure knowledge, perceived norm, personal agency and intention are reported in the trial protocol [44].

### Data collection

Data was collected at baseline and at 4 months post-randomization using questionnaires. At baseline, we collected personal and demographic data and acceptability of at least one method of effective contraception (using the same scales as the primary outcome measure). All baseline data was entered onto the trial database system by the participant on their mobile phone. At 4 month follow-up, we collected all outcomes and the following data: if participants report using an effective method, where they obtained it; current pregnancy intention; whether they knew someone else that took part in the study and if so, if they read each other’s messages; if they stopped the messages; if they experienced physical violence since being in the study and if anything good or bad happened as a result of receiving the messages. An instant message that included a link to the database to complete the follow-up questionnaire was sent to all participants through the app 4 months after downloading the app. If participants did not complete the follow-up questionnaire themselves, local research staff contacted them by telephone to collect their data.

### Sample size

The trial was powered to detect a 15% increase in acceptability of effective contraception in the intervention group compared with the control group. Four hundred and fifty-four participants allowed for 90% power to detect a 15% absolute increase in acceptability, assuming 50% acceptability in the control group (i.e. 50% in the control vs 65% in the intervention, an odds ratio of 1.86). Allowing for 20% loss to follow-up, we aimed to randomize 570 people.

### Statistical analysis

The trial protocol was accepted for publication on 21 July 2017 [44] and the statistical analysis plan was publicly released on 16 August 2017 [45]. The analysis was conducted using Stata 15. Analyses were according to randomized arm and only participants with complete outcome data were included in the principal analysis. All statistical tests were two-sided and considered significant at the 5% level. Unmasking occurred on 29 August 2017, after the analyses outlined within the analysis plan were complete.

### Loss to follow-up and missing data

We used a chi-squared test to investigate whether loss to follow-up differed by arm. We used logistic regression to compare baseline characteristics of participants that completed follow-up against participants that did not. We investigated whether predictors of loss to follow-up differed by arm by testing for an interaction.

### Principal analysis

#### Analysis of the primary outcome

We compared the proportion that reported that at least one method was acceptable in each group using logistic regression. We report the crude and adjusted odds ratio (OR) along with the 95% confidence interval (CI) and *p*-value. We adjusted the primary analysis regression for the following pre-specified baseline covariates: pregnancy intention (wants to avoid/other); gender (female/male); age (16–19/20–24); highest education level completed (university/other) and acceptability of effective contraception (at least one method acceptable/no methods acceptable) [44, 45].

#### Analysis of the secondary outcomes

The analysis of the secondary outcomes was similar to the analysis of the primary outcome. We estimated the difference between the groups using logistic regression and report odds ratios with 95% CIs and *p*-values. Regressions were adjusted for the baseline covariates pregnancy intention, gender, age, education level and acceptability (of at least one method or with acceptability of individual methods, of the corresponding method).

#### Analysis of the process outcomes

The process outcomes perceived norms, personal agency and intention were comprised of ordinal scales. Each scale was analysed individually using ordered logistic regression to estimate proportional ORs. For knowledge, each correct answer received one point. The points were summed and an overall score was produced. We used linear regression to test for a difference in mean scores between the arms. To assess the ‘dose’ of the intervention that the intervention participants received, we analysed the number of messages that participants reported to have read (all, most, some, none) and whether they stopped the messages.

### Additional analyses

#### Sensitivity analyses

We conducted two sensitivity analyses regarding the missing data. In the first, we considered that participants lost to follow-up did not find at least one method acceptable. In the second, we adjusted for the main baseline predictors of missingness. Both sensitivity analyses were adjusted for the baseline covariates pregnancy intention, gender, age, education level and acceptability.

#### Subgroup analysis

We conducted an exploratory subgroup analysis for the primary outcome to determine if the intervention effect varied by baseline characteristics. The pre-specified subgroups were gender (female/male); age (split at the median); marital status (married/not married); number of children (0/1+); ethnicity (Tajik/other); occupation (in education/other); highest education level completed (university/other) and pregnancy intention (wants to avoid/other). Within the subgroups, we assessed heterogeneity of treatment effect with a test for interaction [51–55]. We estimated ORs along with 95% CIs for each subgroup.

#### Contamination

To assess the potential for contamination, we report the proportion of control group participants that reported that they read another participant’s messages and the proportion of intervention participants that reported that their messages were read by another participant.

#### Change from baseline

In addition to the analyses specified in the statistical analysis plan, we tested for a change in the primary outcome from baseline to follow-up, using McNemar’s χ^2^ test for paired data. This post hoc non-randomized analysis was conducted to explore the increase in acceptability overall, as the app included intervention content (see Discussion).

## Results

### Recruitment, randomization, exclusions

Between 16 November 2016 and 1 March 2017, there were 580 randomizations. During the analysis, we discovered that five participants enrolled and were randomized twice. For the three participants that were allocated to the same arm on both randomizations, we kept them in the analysis using the baseline data from their first record. For the two participants that were allocated to different arms, we excluded them from the analysis. This resulted in 573 participants included in the trial (see Discussion).

**Fig. 1:**
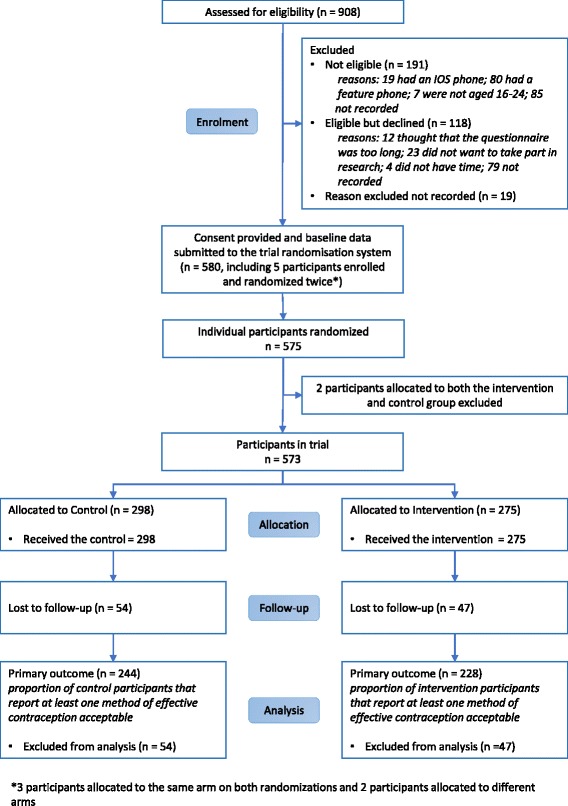
CONSORT diagram

Two hundred and seventy-five participants were allocated to the intervention arm and 298 participants were allocated to the control arm (Fig. 1). No participants withdrew from the trial after allocation.

### Baseline characteristics

Baseline characteristics of trial participants are reported in Table 1. Mean age was 20 years, and 53% were male. Ninety-four percent were not married (259/573), and only 2% (13/573) found at least one method of effective contraception acceptable. Characteristics were similar between the two groups.

**Table 1:** Baseline characteristics

		Control *N* = 298, % (n)	Intervention *N* = 275, % (n)	All participants *N* = 573, % (n)
Age	mean [sd]	20.00 [2.41]	19.93 [2.24]	19.98 [2.33]
	16–19	53.02 (158)	56.73 (156)	54.80 (314)
	20–24	46.98 (140)	43.27(119)	45.20(259)
Gender	female	45.97(137)	47.27 (130)	46.60 (267)
	male	54.03 (161)	52.73 (145)	53.40 (306)
Marital status	married	6.71 (20)	5.82 (16)	6.28 (36)
	not-married	93.29 (278)	94.18 (259)	93.72 (537)
Number of children	0	95.64 (285)	97.09 (267)	96.34 (552)
	1	2.01 (6)	2.18 (6)	2.09 (12)
	2 or more	2.35 (7)	0.73 (2)	1.57 (9)
Ethnicity	Tajik	92.62 (276)	93.82 (258)	93.19 (534)
	Russian	2.35 (7)	0.36 (1)	1.40 (8)
	Uzbek	5.03 (15)	5.45 (15)	5.24 (30)
	other	0 (0)	0.36 (1)	0.17 (1)
Occupation	school	17.79 (53)	17.09 (47)	17.45 (100)
	university	68.46 (204)	70.55 (194)	69.46 (398)
	working	10.74 (32)	10.55 (29)	10.65 (61)
	training	0.67 (2)	0 (0)	0.35 (2)
	parent	0.34 (1)	0 (0)	0.17 (1)
	not working	1.68 (5)	1.82 (5)	1.75 (10)
	university & working	0.34 (1)	0 (0)	0.17 (1)
Highest level of education completed	primary	12.75 (38)	13.09 (36)	12.91 (74)
	secondary	66.11 (197)	59.64 (164)	63.00 (361)
	university	19.46 (58)	25.82 (71)	22.51 (129)
	other	1.68 (5)	1.45 (4)	1.57 (9)
Current pregnancy intention (‘*Do you want a pregnancy now?)*	yes	3.02 (9)	4.00 (11)	3.49 (20)
	no	12.42 (37)	5.82 (16)	9.25 (53)
	unsure	1.01 (3)	0.73 (2)	0.87 (5)
	not married^a^	83.56 (249)	89.45 (246)	86.39 (495)
Baseline method	none	31.88 (95)	29.45 (81)	30.72 (176)
	male condom	2.01 (6)	1.09 (3)	1.57 (9)
	IUD^b^	0.67 (2)	0 (0)	0.35 (2)
	not married^a^	65.10 (194)	69.09 (190)	67.02 (384)
	LAM^c^	0 (0)	0.36 (1)	0.17 (1)
	other	0.34 (1)	0 (0)	0.17 (1)
At least one effective method is acceptable	yes	2.68 (8)	1.82 (5)	2.27 (13)
	no	97.32 (290)	98.18 (270)	97.73 (560)
Pill acceptability	yes	1.34 (4)	0.73 (2)	1.05 (6)
	no	98.66 (294)	99.27 (273)	98.95 (567)
IUD acceptability	yes	1.34 (4)	0 (0)	0.70 (4)
	no	98.66 (294)	100 (275)	99.30 (569)
Injection acceptability	yes	0.67 (2)	1.45 (4)	1.05 (6)
	no	99.33 (296)	98.55 (271)	98.95 (567)
Implant acceptability	yes	0.34 (1)	0.73 (2)	0.52 (3)
	no	99.66 (297)	99.27 (273)	99.48 (570)

^a^The response ‘not married’ was used as a proxy for not being sexually active

^b^*IUD* Intrauterine device

^*c*^*LAM* Lactational amenorrhea method

### Loss to follow-up

Four hundred and seventy-six participants total (83%) contributed follow-up data. Four hundred and seventy-two participants (82%) completed the trial follow-up for the primary outcome (intervention, *n* = 228; control, *n* = 244) (Fig. 1). Retention did not differ between the arms (83% in the intervention vs 82% in the control, *p* = 0.75). The main predictors of retention were male gender (OR 1.78, *p* = 0.01), Tajik ethnicity (OR 2.22, *p* = 0.03) and having completed a level of education lower than university at enrolment (OR 1.79, *p* = 0.02). The effect of these predictors did not differ by arm (interaction test *p*-values: gender, *p* = 0.72; ethnicity, *p* = 0.41; education level, *p* = 0.98). Detailed characteristics of follow-up completers and non-completers are reported in Additional file 1.

### Primary outcome

In the intervention arm, 66% (151/228) reported that at least one method of contraception was acceptable compared to 64% (156/244) in the control arm (Table 2). There was no evidence of a difference in acceptability between the groups (crude OR 1.11, 95% CI .76–1.62, *p* = 0.60; adjusted OR 1.21, 95% CI .80–1.83, *p* = 0.36).

**Table 2:** Primary outcome

	Control *N* = 244, % (n)	Intervention *N* = 228, % (n)	OR (95% CI)	*p*-value
At least one effective method is acceptable^a^	63.93 (156)	66.23 (151)	1.21 (.80–1.83)	0.36

^a^adjusted for pregnancy intention, gender, age, education level and acceptability at baseline

### Secondary outcomes

There were no significant differences in any of the secondary outcomes between the groups (Table 3).

**Table 3:** Secondary outcomes

	Control % (n/N)	Intervention % (n/N)	OR (95% CI)	*p*-value
Use of effective contraception^a^	3.66 (9/246)	1.30 (3/230)	.35 (.06–1.42)	0.18
Pill acceptability^b^	56.56 (138/244)	60.53 (138)	1.32 (.88–2.00)	0.18
IUD acceptability^b^	52.87 (129/244)	51.32 (117/228)	1.00 (.67–1.50)	0.98
Injection acceptability^b^	54.51 (133/244)	55.26 (126/228)	1.14 (.76–1.70)	0.52
Implant acceptability^b^	48.77 (119/244)	48.68 (111/228)	1.08 (.73–1.59)	0.71
Effective contraceptive use during the 4 months^a^	2.88 (7/243)	1.76 (4/227)	.61 (.13–2.42)	0.62
Service uptake^c^ (attended a service one or more times)	10.29 (25/243)	7.93 (18/227)	.76 (.39–1.46)	0.41
Unintended pregnancy^c^	0 (0)	0 (0)	–	–
Induced abortion^c^	0 (0)	0 (0)	–	–

^a^based on unadjusted exact logistic regression, due to small numbers

^b^adjusted for pregnancy intention, gender, age, education level and the corresponding method acceptability at baseline

^c^adjusted for pregnancy intention, gender, age, education level and acceptability at baseline

#### Process outcomes

There were no significant differences in any of the process outcomes between the groups (Table 4).

**Table 4:** Process outcomes

		Control % (n/N)	Intervention % (n/N)	proportional OR^*^ (95% CI), *p*-value
Knowledge of effective contraception		Mean = 4.00 [sd = 2.04]	Mean = 4.08 [sd = 2.02]	.08^**^ (−.29–.44), 0.69
My friends would use the pill, IUD, injection or implant if they wanted to prevent pregnancy	strongly disagree	3.70 (9/243)	1.33 (3/226)	1.40 (.97–2.01), 0.07
	disagree	4.53 (11/243)	5.31 (12/226)	
	not sure	17.28 (42/243)	16.37 (37/226)	
	agree	64.61 (157/243)	59.29 (134/226)	
	strongly agree	9.88 (24/243)	17.70 (40/226)	
My friends would talk to their husband/wife about contraception if they wanted to prevent a pregnancy	strongly disagree	1.23 (3/243)	1.33 (3/226)	1.09 (.76–1.57), 0.64
	disagree	5.35 (13/243)	6.64 (15/226)	
	not sure	16.05 (39/243)	15.93 (36/226)	
	agree	65.02 (158/243)	59.29 (134/226)	
	strongly agree	12.35 (30/243)	16.81 (38/226)	
If you wanted to use the pill, IUD, injection or implant, how easy would it be for you to use it? (women only)	very difficult	7.62 (8/105)	5.83 (6/103)	1.43 (.87–2.34), 0.16
	difficult	17.14 (18/105)	9.71 (10/103)	
	not sure	27.62 (29/105)	29.13 (30/103)	
	easy	38.10 (40/105)	43.69 (45/103)	
	very easy	9.52 (10/105)	11.65 (12/103)	
If you wanted to talk to your husband/wife about contraception, how easy would it be for you to talk to him/her?	very difficult	3.70 (9/243)	3/10 (7/226)	1.22 (.86–1.73), 0.26
	difficult	6.17 (15/243)	7.52 (17/226)	
	not sure	14.81 (36/243)	14.16 (32/226)	
	easy	60.49 (147/243)	53.10 (120/226)	
	very easy	14.81 (36/243)	22.12 (50/226)	
If you wanted to use the pill, IUD, injection or implant, how certain are you that you could use it? (women only)	very certain I could not	2.86 (3/105)	5.83 (6/103)	.99 (.60–1.63), 0.96
	certain I could not	6.67 (7/105)	7.77 (8/103)	
	not sure	38.10 (40/105)	32.04 (33/103)	
	certain I could	40.00 (42/105)	41.75 (43/103)	
	very certain I could	12.38 (13/105)	12.62 (13/103)	
If you wanted to talk to your husband/wife about contraception, how certain are you that you could talk to him/her?	very certain I could not	1.23 (3/243)	2.65 (6/226)	1.10 (.78–1.53), 0.60
	certain I could not	13.17 (32/243)	12.39 (28/226)	
	not sure	16.46 (40/243)	16.81 (38/226)	
	certain I could	50.62 (123/243)	44.25 (100/226)	
	very certain I could	18.52 (45/243)	23.89 (54/226)	
I intend to use the pill, IUD, injection or implant	strongly disagree	4.76 (5/105)	2.91 (3/103)	1.37 (.84–2.25), 0.21
	disagree	10.48 (11/105)	12.62 (13/103)	
	not sure	31.43 (33/105)	25.24 (26/103)	
	agree	39.05 (41/105)	34.95 (36/103)	
	strongly agree	14.29 (15/105)	24.27 (25/103)	
Number of messages read	all		32.16 (73/227)	
	most		43.61 (99/227)	
	some		18.50 (42/227)	
	none		5.73 (13/227)	
Proportion of intervention participants that stopped the intervention			29.07 (66/227)	

^*^estimated from ordered logistic regression

^**^mean difference

#### Potential for contamination

Three percent (8/243) of control participants said that they read the messages of someone else in the study. Nine percent (21/227) of intervention participants said that someone else in the study read their messages.

#### Participants’ report of physical violence during the study

Overall, 0.85% (4/470) reported that they experienced physical violence since being in the study (0.41% in the control and 1.32% in the intervention, *p* = 0.57).

### Sensitivity analyses

The effect of the intervention on the primary outcome observed in the principal analysis did not change when we considered participants lost to follow-up did not find at least one method acceptable (OR 1.20, 95% CI .84–1.73, *p* = 0.31) or when we adjusted the model for the predictors of missingness (OR 1.21, 95% CI .80–1.85, *p* = 0.35).

### Subgroup analysis

**Fig. 2:**
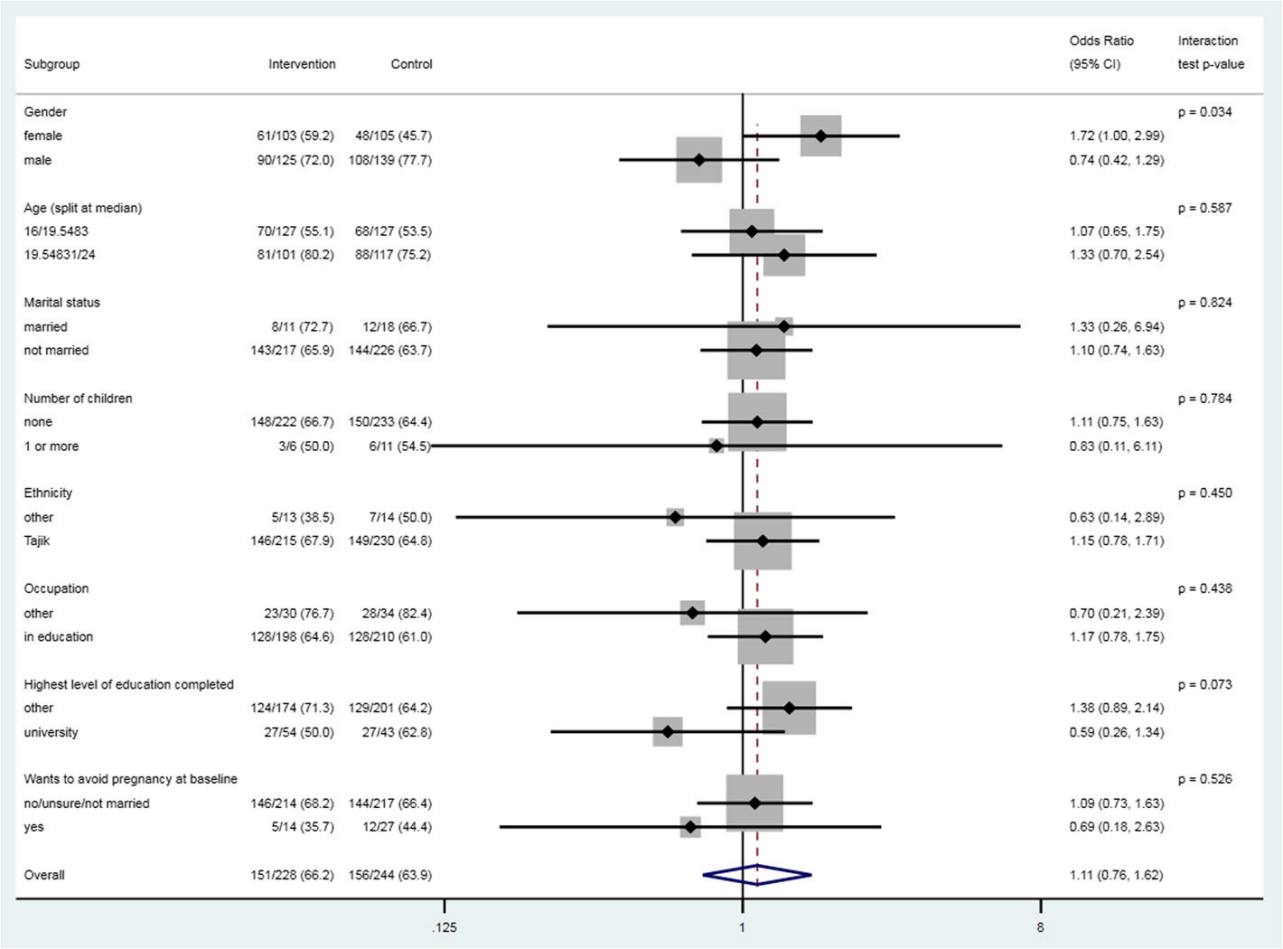
Primary outcome by pre-specified subgroups

There was some evidence that the effect of the intervention was greater among women compared to men (interaction test *p* = 0.03). (Fig. 2).

### Change from baseline analysis

**Fig. 3:**
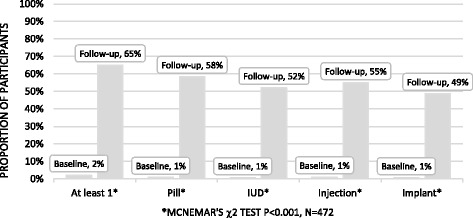
Method acceptability at baseline and follow-up

Among the 472 participants who completed follow-up 2% (*n* = 10) thought that at least one method was acceptable at baseline, which increased to 65% at follow-up (*n* = 307, *p* < 0.001) (Fig. 3). Acceptability for the individual methods increased from 1% at baseline to 49%–58% at follow-up (*p* < 0.001).

## Discussion

### Main results

Contrary to what was planned in the trial protocol, the app contained intervention content. Both intervention and control participants received intervention content targeting knowledge and attitudes towards effective contraception, including the BCM ‘belief selection’. The trial therefore evaluated the effect of the whole intervention with all ten BCMs (belief selection, facilitation, anticipated regret, guided practice, verbal persuasion, tailoring, cultural similarity, arguments, shifting perspective and goal setting) delivered by instant messaging, compared to a proportion of the intervention delivered on the app pages with the BCM belief selection.

The trial found no evidence of a difference in acceptability of at least one effective contraceptive method between the intervention and control groups. There was also no evidence of a difference in any of the secondary and process outcomes between the groups (use of effective contraception, service uptake, knowledge, perceived norms, personal agency and intention to use effective contraception). This indicates that the intervention content delivered by the intervention messages only (includes nine additional BCMs targeting attitudes and personal agency) did not have an additional benefit over the app regarding these outcomes. The subgroup analysis suggests that the intervention delivered by instant messaging could be more effective among women compared to men. When data from both groups were analysed together, there was a large statistically significant increase in acceptability from baseline to follow-up.

### Comparisons with other research

Trials that have evaluated interventions delivered by mobile phone to improve contraceptive-related outcomes have had mixed results [33–43]. We are conducting trials in Bolivia and Palestine that are evaluating the effect of interventions similar to the Tajik intervention on acceptability and use of effective contraception [56, 57]. The results of the three trials together should contribute to a better understanding of the effect of the intervention evaluated in this Tajik trial.

Our trial shows no additional benefit on the outcomes from the nine BCMs deliver by instant messaging. No previous research reports the effectiveness of these BCMs aimed at improving contraceptive-related outcomes delivered by mobile phone [58].

Ongoing trials of interventions delivered by mobile phone to improve reproductive health are measuring participants’ experience of violence during their participation in the trial [56, 57, 59]. In this Tajik trial, we found no association between the intervention and experience of violence. While this is reassuring, both groups had access to the app so we are unable to assess the effect of the app on partner violence.

### Strengths and limitations

The trial conduct has a number of strengths. We recruited our target number of participants and were able to collect follow-up data for an acceptable proportion of them, given that the sample size allowed for 20% loss. We developed and tested a remote trial database and randomization system, which successfully generated and concealed the allocation sequence and achieved well-balanced groups. An important limitation is that the app included intervention content, as discussed above. This constitutes a protocol deviation and the trial was therefore not able to answer the primary question it aimed to answer. Because the self-reported acceptability scales were collected by telephone by the research staff, participants may have been more likely to report positive attitudes than they were at baseline where they completed the questionnaire by themselves on their phones. Regarding the large increase in acceptability from baseline to follow-up, we cannot rule out the possibility that at least a portion of this increase was due to participation in the trial as opposed to the intervention itself; participants were aware that the trial involved changing attitudes towards contraception. Five participants enrolled and were randomized twice.

There were inconsistencies in participants’ self-reporting of marital status. The proportion that responded ‘not married’ to the current pregnancy intention (495/573, 86%) and the baseline method question (384/573, 67%) is lower than the proportion that responded ‘not married’ when asked directly about their marital status (537/573, 94%). We cannot say why these inconsistencies occurred. However, we can speculate that some participants who responded ‘not married’ to the marital status question were sexually active and responded to the other two questions with responses other than ‘not married’.

Thirty six percent of people assessed for eligibility (328/908) were excluded from the study. The reason for ineligibility was not recorded for 85 people, which could limit the generalizability of the trial findings. While the recording of this information was not complete, of those that are known, the majority appear to have been excluded because they either did not have an Android phone (*n* = 99). If those who do not own a smartphone are less likely to find at least one method of effective contraception acceptable, this could affect the generalisability of the results. Smartphone ownership is rapidly increasing however, and ownership could be an option for a greater proportion of young people across different socioeconomic communities in the near future.

### Implications of the findings

The finding that the intervention instant messages did not have an additional benefit over the app along with the large increase in acceptability from baseline to follow-up suggests that participants read the app contraception pages. It may be that in a context such as Tajikistan, where young people have limited access to information and support about reproductive health, they are willing to read static app pages about this topic. In comparison, a trial in the United Kingdom found that young people did not engage heavily with a sexual and reproductive health website [60, 61]. In contexts such as the United Kingdom where information and support are more accessible, interventions delivered on app pages and websites may be utilized less frequently than in contexts such as Tajikistan.

Because the intervention content included on the app aimed to improve knowledge of and attitudes towards effective contraception, it is not surprising that there was no evidence of a difference between the groups regarding these outcomes. Though the large increase in acceptability from baseline to follow-up cannot be unequivocally attributed to the intervention content, an increase this large suggests that the intervention content included on the app at least was partially effective in improving attitudes towards the effective methods. Because the intervention is well-specified, we were able to identify the components of the intervention that may have been effective in producing this change (accurate information and targeting beliefs using the BCM belief selection) [46, 48].

Despite the contamination that occurred, intervention participants received content that control participants did not. The secondary outcomes use and service uptake and the process outcomes personal agency and intention are related to the content that only intervention participants received. There are a number of potential explanations for why we did not observe a difference between the groups in these outcomes. The first is that the BCMs targeting these outcomes did not work. This could have been because the conditions under which the methods have been shown to be effective were not fully satisfied [46, 48]. In addition, because a large proportion of meaning comes from visual cues in face-to-face interaction [46], some of the meaning of the BCMs may have been lost when delivered by mobile phone. For example, the BCM ‘guided practice’ requires skill demonstration, enactment and individual feedback. While the intervention messages demonstrated and provided instruction, we were not able to observe the participant enacting the behavior or to provide individual feedback. This may have resulted in a loss of effectiveness of the BCM. Another explanation is that intervention could be more effective on these secondary and process outcomes with people where the behaviour is salient, such as with those who are married/sexually active or soon to be. In this trial however, only 6% (36/573) were married/sexually active, which was too small to explore this possibility. Alternatively, the app alone may have been effective in influencing these secondary and process outcomes; in the Tajik context, providing accurate information from a credible source and targeting the pre-identified beliefs may be sufficient. Finally, these secondary and process outcomes could have be so strongly influenced by environmental conditions (e.g. stigma regarding sexual activity before marriage and pressure to bear children) that they are not amenable to change by a mobile phone intervention only.

While caution is necessary in interpreting the results of the subgroup analysis, it suggests that the whole intervention delivered by instant messaging could be more effective among women compared to men. The trials in Bolivia and Palestine involve women only so the results should provide additional evidence of the intervention’s effectiveness in women.

We are currently conducting qualitative interviews with trial participants to explore their experiences in receiving the intervention and app content. If participants were positive about receiving the intervention messages, this could support the delivery of the messages with the download of the app. The fact that the intervention is already developed and therefore inexpensive to deliver, plus the fact that it does not appear to cause harm, also supports the delivery of the messages with the download of the app.

## Conclusions

This trial demonstrated that the whole intervention delivered by app instant messaging provided no additional benefit over a portion of the intervention delivered by the app pages. An analysis of participants randomized to the control and intervention groups together showed a large significant increase in acceptability from baseline to follow-up. Further research is needed to establish the effect of the intervention on attitudes towards and use of effective contraceptive methods among married/sexually active young people.

## Additional file

Additional file 1 Baseline data by follow-up status. Baseline characteristics by follow-up completion. The baseline characteristics of the participants that completed the primary outcome and the baseline characteristics of participants that did not complete the primary outcome. (DOCX 17 kb)

## Abbreviations

BCM: Behaviour change method
CI: Confidence interval
IPPF: International Planned Parenthood Federation
LSHTM: London School of Hygiene & Tropical Medicine
OR: Odds ratio
TFPA: Tajik Family Planning Association
