# Correction to: A randomized controlled trial of an intervention delivered by mobile phone app instant messaging to increase the acceptability of effective contraception among young people in Tajikistan

**DOI:** 10.1186/s12978-018-0496-5

**Published:** 2018-03-26

**Authors:** Ona McCarthy, Irrfan Ahamed, Firuza Kulaeva, Ravshan Tokhirov, Salokhiddin Saibov, Marieka Vandewiele, Sarah Standaert, Baptiste Leurent, Phil Edwards, Melissa Palmer, Caroline Free

**Affiliations:** 1 Department of Population Health, Faculty of Epidemiology and Population Health, London School of Hygiene & Tropical Medicine, Keppel Street, London, WC1E 7HT UK; 2 Tajik Family Planning Association, 10 Rudaki Avenue, TC ‘Sadbarg’, 7th floor, Dushanbe, Tajikistan; 3 International Planned Parenthood Federation European Network, Rue Royale 146, 1000 Brussels, Belgium; 4 Department of Medical Statistics, Faculty of Epidemiology and Population Health, London School of Hygiene & Tropical Medicine, Keppel Street, London, WC1E 7HT UK

## Correction

After publication of the original article [1], it came to the authors’ attention that there is a typo in the Results section. The following section should have read as follows:

**Participants’ report of physical violence during the study.**

Overall, 0.85% (4/470) reported that they experienced physical violence since being in the study (0.41% in the control and 1.32% in the intervention, **Pearson’s chi2** ***p*** = **0.28**).
