# Supplementary material for: A randomized controlled trial of an intervention delivered by mobile phone app instant messaging to increase the acceptability of effective contraception among young people in Tajikistan
Source: Reprod Health. 2018 Feb 13;15:28. doi: 10.1186/s12978-018-0473-z (PMC5809875; doi:10.1186/s12978-018-0473-z)
Supplement: Supplementary file 1 — Baseline data by follow-up status. Baseline characteristics by follow-up completion. The baseline characteristics of the participants that completed the primary outcome and the baseline characteristics of participants that did not complete the primary outcome. (DOCX 17 kb) [file 12978_2018_473_MOESM1_ESM.docx]

| **Baseline characteristics** | | **Primary outcome completers** | | **Primary outcome non-completers** | | **All participants** | |
| --- | --- | --- | --- | --- | --- | --- | --- |
|  |  | **Control**  N = 244  % (n) | **Intervention**  N = 228  % (n) | **Control**  N = 54  % (n) | **Intervention**  N = 47  % (n) | **Control**  N = 298  % (n) | **Intervention**  N = 275  % (n) |
| Age | mean [sd] | 20 [2.44] | 20 [2.35] | 19.73 [2.29] | 19.80 [1.65] | 20.00 [2.41] | 19.93 [2.24] |
|  | 16-19 | 52.05 (127) | 55.70 (127) | 57.41 (31) | 61.70 (29) | 53.02 (158) | 56.73 (156) |
|  | 20-24 | 47.95 (117) | 44.30 (101) | 42.59 (23) | 38.30 (18) | 46.98 (140) | 43.27(119) |
| Gender | female | 43.03 (105) | 45.18 (103) | 59.26 (32) | 57.45 (27) | 45.97(137) | 47.27 (130) |
|  | male | 56.97 (139) | 54.82 (125) | 40.74 (22) | 42.55 (20) | 54.03 (161) | 52.73 (145) |
| Marital status | married | 7.38 (18) | 4.82 (11) | 3.70 (2) | 10.64 (5) | 6.71 (20) | 5.82 (16) |
|  | not-married | 92.62 (226) | 95.18 (217) | 96.30 (52) | 89.36 (42) | 93.29 (278) | 94.18 (259) |
| Number of children | 0 | 95.49 (233) | 97.37 (222) | 96.30 (52) | 95.74 (45) | 95.64 (285) | 97.09 (267) |
|  | 1 | 2.05 (5) | 1.75 (4) | 1.85 (1) | 4.26 (2) | 2.01 (6) | 2.18 (6) |
|  | 2 or more | 2.46 (6) | 0.88 (2) | 1.85 (1) | 0 (0) | 2.35 (7) | 0.73 (2) |
| Ethnicity | Tajik | 94.26 (230) | 94.30 (215) | 85.19 (46) | 91.49 (43) | 92.62 (276) | 93.82 (258) |
|  | Russian | 2.05 (5) | 0.44 (1) | 3.70 (2) | 0 (0) | 2.35 (7) | 0.36 (1) |
|  | Uzbek | 3.69 (9) | 5.26 (12) | 11.11 (6) | 6.38 (3) | 5.03 (15) | 5.45 (15) |
|  | other | 0 (0) | 0 (0) | 0 (0) | 2.13 (1) | 0 (0) | 0.36 (1) |
| Occupation | school | 16.39 (40) | 17.54 (40) | 24.07 (13) | 14.89 (7) | 17.79 (53) | 17.09 (47) |
|  | university | 68.85 (168) | 69.30 (158) | 66.67 (36) | 76.60 (36) | 68.46 (204) | 70.55 (194) |
|  | working | 11.89 (29) | 12.28 (28) | 5.56 (3) | 2.13 (1) | 10.74 (32) | 10.55 (29) |
|  | training | 0.41 (1) | 0 (0) | 1.85 (1) | 0 (0) | 0.67 (2) | 0 (0) |
|  | parent | 0.41 (1) | 0 (0) | 0 (0) | 0 (0) | 0.34 (1) | 0 (0) |
|  | not working | 1.64 (4) | 0.88 (2) | 1.85 (1) | 6.38 (3) | 1.68 (5) | 1.82 (5) |
|  | university & working | 0.41 (1) | 0 (0) | 0 (0) | 0 (0) | 0.34 (1) | 0 (0) |
| Highest level of education completed | primary | 12.70 (31) | 13.16 (30) | 12.96 (7) | 12.77 (6) | 12.75 (38) | 13.09 (36) |
|  | secondary | 68.44 (167) | 62.28 (142) | 55.56 (30) | 46.81 (22) | 66.11 (197) | 59.64 (164) |
|  | university | 17.62 (43) | 23.68 (54) | 27.78 (15) | 36.17 (17) | 19.46 (58) | 25.82 (71) |
|  | other | 1.23 (3) | 0.88 (2) | 3.70 (2) | 4.26 (2) | 1.68 (5) | 1.45 (4) |
| Current pregnancy intention  (‘*Do you want a pregnancy now?)* | yes | 3.69 (9) | 3.07 (7) | 0 (0) | 8.51 (4) | 3.02 (9) | 4.00 (11) |
|  | no | 11.07 (27) | 6.14 (14) | 18.52 (10) | 4.26 (2) | 12.42 (37) | 5.82 (16) |
|  | unsure | 0.41 (1) | 0.44 (1) | 3.70 (2) | 2.13 (1) | 1.01 (3) | 0.73 (2) |
|  | not married^*^ | 84.84 (207) | 90.35 (206) | 77.78 (42) | 85.11 (40) | 83.56 (249) | 89.45 (246) |
| Baseline method | none | 28.28 (69) | 25.88 (59) | 48.15 (26) | 46.81 (22) | 31.88 (95) | 29.45 (81) |
|  | male condom | 1.64 (4) | 1.32 (3) | 3.70 (2) | 0 (0) | 2.01 (6) | 1.09 (3) |
|  | IUD^**^ | 0.82 (2) | 0 (0) | 0 (0) | 0 (0) | 0.67 (2) | 0 (0) |
|  | not married^*^ | 68.85 (168) | 72.37 (165) | 48.15 (26) | 53.19 (25) | 65.10 (194) | 69.09 (190) |
|  | LAM^***^ | 0 (0) | 0.44 (1) | 0 (0) | 0 (0) | 0 (0) | 0.36 (1) |
|  | other | 0.41 (1) | 0 (0) | 0 (0) | 0 (0) | 0.34 (1) | 0 (0) |
| At least one effective method is acceptable | yes | 2.46 (6) | 1.75 (4) | 3.70 (2) | 2.13 (1) | 2.68 (8) | 1.82 (5) |
|  | no | 97.54 (238) | 98.25 (224) | 96.30 (52) | 97.87 (46) | 97.32 (290) | 98.18 (270) |
| Pill acceptability | yes | 1.23 (3) | 0.44 (1) | 1.85 (1) | 2.13 (1) | 1.34 (4) | 0.73 (2) |
|  | no | 98.77 (241) | 99.56 (227) | 98.15 (53) | 97.87 (46) | 98.66 (294) | 99.27 (273) |
| IUD acceptability | yes | 1.23 (3) | 0 (0) | 1.85 (1) | 0 (0) | 1.34 (4) | 0 (0) |
|  | no | 98.77 (241) | 100 (228) | 98.15 (53) | 100 (47) | 98.66 (294) | 100 (275) |
| Injection acceptability | yes | 0.82 (2) | 1.32 (3) | 0 (0) | 2.13 (1) | 0.67 (2) | 1.45 (4) |
|  | no | 99.18 (242) | 98.68 (225) | 100 (54) | 97.87 (46) | 99.33 (296) | 98.55 (271) |
| Implant acceptability | yes | 0.41 (1) | 0.88 (2) | 0 (0) | 0 (0) | 0.34 (1) | 0.73 (2) |
|  | no | 99.59 (243) | 99.12 (226) | 100 (54) | 100 (47) | 99.66 (297) | 99.27 (273) |

^*^The response ‘not married’ was used as a proxy for not being sexually active

^**^IUD = intrauterine device

^***^LAM = Lactational amenorrhea method
